# Age- and Gender-Specific Components of the Metabolic Syndrome in 2228 First Graders: The PEP Family Heart Study

**DOI:** 10.1155/2013/394807

**Published:** 2013-02-17

**Authors:** Peter Schwandt, Thomas Bertsch, Evelyn Liepold, Gerda-Maria Haas

**Affiliations:** ^1^Arteriosklerose-Präventions-Institut, München-Nürnberg, 81477 München, Germany; ^2^Ludwig Maximilians Universität München, 80539 München, Germany; ^3^Institut für Klinische Chemie, Laboratoriumsmedizin und Transfusionsmedizin-Zentrallaboratorium, Klinikum Nürnberg, 90419 Nürnberg, Germany

## Abstract

Because first graders are critical for excess weight gain, we assessed components of the metabolic syndrome (MetS) using the pediatric definition of the International Diabetes Federation (IDF). We compared four MetS components as defined by the IDF with age- and gender-specific components in 2228 first graders at the age of 6. The growth curves were derived from 22113 children and adolescents who participated in the PEP Family Heart Study. The aim was to determine in first graders precise values of waist circumference (WC), blood pressure (BP), triglycerides (TG), and HDL-Cholesterol (HDL-C) based on growth curves that were developed for a large German population of youths and to assess the prevalence in terms of both definitions at this critical age. The prevalence of high blood pressure for age was 13% compared with only 2% according to IDF. Because of this considerable divergence, we propose to define MetS components based on national growth curves.

## 1. Introduction

There is still no universally accepted definition of the metabolic syndrome (MetS) in children and adolescents because of heterogeneous criteria as adult cut offs, ignoring the effects of growth, and lack of cut points for waist circumference (WC) and for lipid levels throughout youth [1]. The International Diabetes Federation (IDF) defined MetS criteria for children and adolescents limiting their full application to age from 10 to <16 years [2]. Since the prevalence of MetS in children varies widely, its single components should be recognized as early as possible [1]. Early school years between 1st grades and 3rd grades are critical for excess weight gain since children who have MetS do increase their risk of cardiovascular disease (CVD) in adulthood [3, 4].

Because the IDF does not recommend measuring MetS in children who are younger than 10 years old, our aim was to characterize four of the MetS components in terms of absolute values derived from age- and gender-specific growth curves in a large sample of 6-year old first graders. Furthermore, we compared the prevalence of four IDF components as age- and gender-specific values.

## 2. Subjects and Methods

We investigated 2228 German first graders (1116 boys and 1112 girls, median age 6.0 years) who participated in yearly cross-sectional surveys (1994−2003) of the Prevention Education Program (PEP) Family Heart Study. Continuously trained research assistants measured WC, systolic (SBP) and diastolic (DBP) blood pressure, using an oscillometric BP-measuring device, fasting triglycerides (TG), and high-density-cholesterol (HDL-C) as previously described [7–10]. For the traditional definition we used the IDF cutoffs for the four MetS components in terms of WC ≥ 90th percentile, SBP ≥ 130 and/or DBP ≥ 85 mm Hg, TG ≥ 1.7 mmol/L, and HDL-C ≤ 1.03 mmol/L [4]. We derived age- and gender-specific values from percentiles of 22113 youths aged 3–18 years participating in the PEP Family Heart Study using the LMS Chartmaker Pro, version 2.3, estimating the skewness parameter *L*, the median *M*, and a measure of variation *S*, and excluded outlying values <3rd and >97th percentiles by winsorization [11]. We defined age- and- gender-specific cut-off values as WC ≥ 90th percentile, SBP and DBP ≥ 95th percentile, TG ≥ 95th percentile, and HDL-C ≤ 5th percentile. For statistical analyses, we used SPSS 18.0 and developed age- and gender-specific growth curves using the LMS method [12]; *P* < 0.05 was considered significant.

## 3. Results and Discussion

The aim of the current study was to develop for 6-year-old first graders age- and gender-specific MetS components and to compare their prevalence with the IDF definitions.  Table 1 presents mean values of the four components demonstrating significantly higher values for WC and HDL-C and lower TG values in boys than in girls. 

Blood pressure is the only MetS component presenting worldwide-accepted age- and gender-specific values for children and adolescents [5]. Therefore, we compared age- and gender-specific and height-adjusted blood pressure values at the 95th percentile for 6-year-old first graders of the current study and of another representative German study [6] resulting in nearly identical BP values (Table 2). The mean BP in 6-years old children in the three studies [5–7] is 113/75 mmHg for both genders whereas the IDF recommends 130/85 mmHg for the age range 10 to <16 years excluding younger age groups because of insufficient data [2]. However, because the BP database has increased worldwide since 2007 we propose establishing age- and gender-specific recommendations for ages 6 to <10 years instead of excluding this important age.

**Figure 1:**
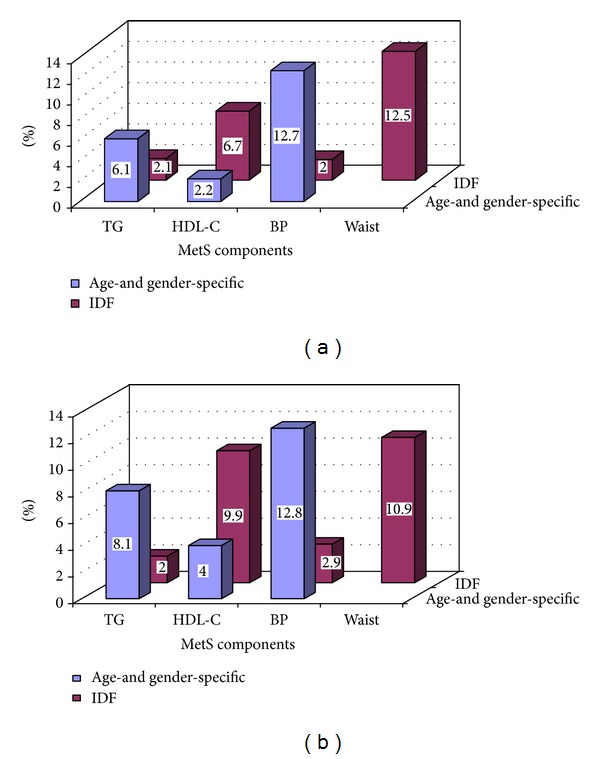
(a) Prevalence of four components of the metabolic syndrome in male first graders depending on IDF, respectively, age- and gender-specific growth curves. (b) Prevalence of four components of the metabolic syndrome in female first graders depending on IDF, respectively, age- and gender-specific growth curves.

However, the IDF recommends assessing MetS components only if there is a positive family history regarding MetS, diabetes, dyslipidemia, hypertension, CVD, and obesity [2]. Applying IDF recommendations, we found a prevalence of hypertension of 2.0% for boys and 2.9% for girls compared with 12.7% for boys and 12.8% for girls based on age- and gender-specific values (Figure 1). However, there are no worldwide-standardized reference values for low HDL-C, elevated TG and WC in children.

The Third Report of the Adults Treatment Panel (ATP III) defined the pediatric MetS components in terms of fasting plasma glucose (FPG) ≥ 6.1 mmol/L, TG ≥ 1.1 mmol/L, HDL-C ≤ 1.3 mmol/L, WC > 75th percentile, and SBP > 90th percentile for gender, age, and height [13]. The pediatric IDF definition of MetS was used in studies performed in the USA and in Europe [14, 15], thirteen pediatric studies using modified ATP III and WHO criteria [1]. In Iranian adolescents population-derived age-and gender-specific cutoffs were calculated for WC and blood pressure (BP) but not for TG and HDL-C [16]. Age-specific percentile values were described for WC, TG, and HDL-C for 6–11 years old Iranian and German children demonstrating considerable ethnic differences [17]. For adolescents age-specific cut points and the corresponding percentiles for males and females using data from the National Health and Nutrition Examination Surveys from 1988 to 2002 were described [18].

## 4. Conclusions

School enrollment and first grade are important assessing pediatric cardiometabolic risk factors. However, current definitions of single components of pediatric MetS exclude first graders. As demonstrated in this study, 13% had hypertension based on age- and gender-specific values instead of 2% using IDF definition for hypertension. Since the use of multiple definitions of the metabolic syndrome argues strongly for the development of a standard pediatric definition [19], we propose national age- and gender-specific definitions for blood pressure and body mass index as they have been realized worldwide [5, 20].

## Figures and Tables

**Table 1:** Components of the metabolic syndrome in 2228 first graders; mean (SD).

	Boys (*n*)		Girls (*n*)	
Mean age (y) (SD)	1116	6.4 (.2)	1112	6.4 (0.2)
Median	1116	6.0	1112	6.0
WC (cm) (SD)	1095	57* (5.2)	1108	56 (5.2)
SBP mmHg (SD)	1080	104.1 (8.8)	1094	103.9 (8.9)
DBP mmHg (SD)	1080	68.2 (8.3)	1094	68.4 (8.3)
Triglycerides (mmol/L) (SD)	506	0.70 (0.32)	519	0.78* (0.33)
HDL-C (mmol/L) (SD)	493	1.54 (0.35)	505	1.49* (0.36)

*P < 0.05 indicates significance between genders.

**Table 2:** Comparison of blood pressure recommendations from IDF [2] for 10–<16 year-old children and for 6-year-old children at the 95th percentile from the Fourth Report [5], KIGGS [6] and PEP [7].

	Boys	Girls
	SBP/DBP	SBP/DBP
IDF 10–<16 years	130/85 mm Hg	130/85 mm Hg
6-year-old 95th percentile		
PEP [7]	115/79 mm Hg	115/79 mm Hg
4th report [5]	114/74 mm Hg	111/74 mm Hg
KIGGS [6]	111/71 mm Hg	112/72 mm Hg
Mean	**113/75** mm Hg	**113/75** mm Hg
